# Book Reviews

**Published:** 1809-09

**Authors:** 


### CRITICAL ANALYSIS
OF THE
### RECENT PUBLICATIONS
ON THE
### DIFFERENT BRANCHES OF PHYSIC, SURGERY, AND MEDICAL PHILOSOPHY.

*Medico-Chirurgical Transactions, published by the Medical and Chirurgical Society of London. Vol. I.*

( Concluded from our last, pp. 152—166. )

ARTICLE 19.—*Observations on the Distemper in Dogs.* By EDWARD JENNER, M. D. F. R. S.

This is a truly valuable paper, inasmuch as it describes a canine epizootic with great accuracy from its commencement through all its forms, with the appearances on dissection. The disease begins principally with inflammation in the substance of the lungs, and all the mucous membrane connected with it, even to the nostrils, and the membranes lining the bones. These are sometimes so inflamed as to produce extravasation of blood, or rather effusion, with coagulation on the surface.

"The breathing is short and quick, and the breath is often fetid. The teeth are covered with dark looking mucus. There is frequently a vomiting of a glary fluid. The dog commonly refuses food, but his thirst seems insatiable, and nothing seems to cheer him like the sight of water. The bowels, though generally constipated as the disease advances, are frequently affected with diarrhœa at its commencement. The eyes are inflamed; and the sight is often obscured by mucus secreted from the eye-lids, or by the opacity of the cornea. The brain is often affected as early as the second day after the attack. The animal becomes stupid, and his general habits are changed. In this state, if not prevented by loss of strength, he sometimes wanders from his home. He is frequently endeavouring to expel, by forcible expirations, the mucus from the trachea and fauces, with a peculiar rattling noise. His jaws are generally smeared with it, and it sometimes flows out in a frothy state, from his frequent champing. During the progress of the disease, especially in its advanced stages, he is disposed to bite and gnaw any thing within its reach. He has sometimes epileptic fits, or quick successions of general, though slight convulsive spasms of the muscles. If the dog survives, this affection of the muscles continues through life. He is often attacked with fits of a different description. He first staggers, then tumbles, rolls, cries as if whipped, and tears up the ground with his teeth and fore-feet. He then lies down senseless and exhausted. On recovering he gets up, moves his tail, looks placid, comes to a whistle, and appears in every every respect much better than before the attack. The eyes, during this paroxysm, look bright, and unless previously rendered dim by mucus, or opacity of the cornea, seem as if they were starting from the sockets. He becomes emaciated, and totters from feebleness in attempting to walk, or from a partial paralysis of the hind legs. In this state he sometimes lingers on till the third or fourth week, and then either begins to shew signs of returning health (which seldom happens when the symptoms have continued with this degree of violence) or expires. During convalescence, he has sometimes, though rarely, profuse hæmorrhage from the nose. When the inflammation of the lungs is very severe, he frequently dies on the third day. I knew one instance of a dog's dying within twenty-four hours after the seizure, and in that short space of time the greater portion of the lungs was, from exudation, converted into a substance nearly as solid as the liver of a sound animal. In this case, the liver itself was considerably inflamed, and the eyes and flesh universally were tinged with yellow, though I did not observe any thing obstructing the biliary ducts. In other instances, I have also observed the eyes looking yellow.

"The above is a description of the disease in its severest form; but in this, as in the diseases of the human body, there is every gradation in its violence. There is also another affinity to some human diseases, viz. that the animal which has once gone through it, very rarely meets with a second attack. Fortunately, this distemper is not communicable to man. Neither the effluvia from the diseased dog, nor the bite, have proved in any instance infectious; but as it has often been confounded with canine madness, as I have before observed, it is to be wished that it were more generally understood; for those who are bitten by a dog in this state, are sometimes thrown into such perturbation, that hydrophobic symptoms have actually arisen from the workings of the imagination. Mr. John Hunter used to speak of a case somewhat of this description in his lectures.* Having never, to a certainty, seen a dog with hydrophobia, I am of course unable to lay down a positive criterion for distinguishing between that disease and the distemper, in the precise way I could wish; but if the facts have been correctly stated, that in hydrophobia the eye of the dog has more than ordinary vivacity in it, and as the term implies, he refuses to take water, and shudders even at the sight of it, while in the distemper he

'* A gentleman who received a severe bite from a dog, soon after fancied the animal was mad. He felt a horror at the sight of liquids, and was actually convulsed on attempting to swallow them. So uncontrolable were his prepossessions, that Mr. Hunter conceived he would have died, had not the dog which inflicted the wound been fortunately found and brought into his room in perfect health. This soon restored his mind to a state of tranquillity. The sight of water no longer affected him, and he quickly recovered.'

looks dull and stupid, is always seeking after water, and never satisfied with what he drinks, there can be no loss for a ready discriminating line between the two diseases."

Such is this valuable record, for which we are under many obligations to Dr. Jenner. But we are not satisfied in three points of no small importance. First, as to the name; though we doubt not, among the farriers in Glocestershire, the disease may be called *The Distemper*, yet we suspect the same name is given to many other canine diseases in other parts of England, and perhaps even in Glocestershire itself. We have seen young dogs about the period of their advancement to puberty, with all the symptoms of epilepsy recurring very frequent. This has been called by the breeders "the Distemper," though it was neither contagious, nor attended with any pulmonary symptom, unless a foaming of the mouth can be called such. We mention this, not to undervalue Dr. Jenner's communication, but to induce him and other philosophers to study, with the diseases of animals, the vernacular names given to them in different parts of the kingdom.*

Another objection we have, is, to the certainty with which the author assures us, that the disease resembles those contagions to which the human race is liable, viz. "that the animal which has once gone through it *very rarely* meets with a second attack." This fact should be better ascertained before we determine that the disease here described is any thing more than the canine *febris carcerum*, perhaps determined to those particular organs by some constitution of the atmosphere.

Our third objection is, to the diagnostic distinction between this disease and rabies. It is but justice to Dr. Jenner to repeat his remark, that having never seen a dog with hydrophobia, he cannot lay down a positive distinguishing criterion. But it is well known, that in some dogs, whose bite has induced rabies, no difficulty of swallowing, nor disgust at the sight of water, has occurred.

ARTICLE 20.—*Two Cases of Small-pox Infection communicated to the Fœtus in Utero, under particular Circumstances, with additional Remarks.* BY EDWARD JENNER, M. D. F. R. S.

In the first of these Cases, the mother having previously passed through small-pox, was, a few days before her delivery, exposed to the sight and smell of a most disgusting subject under that disease. The child, when born, was free from disease, but on the 5th day after became indisposed, and on the 7th variolous pustules appeared, which maturated in the usual way. The mother was no way indisposed.

"This case," says Dr. Jenner, "decidedly proves, that the small-pox virus may affect the human frame, even to its inmost recesses,

---

* We shall feel particularly thankful if any country gentleman, whether practitioner or not, will favour us with communications on these subjects.

recesses, although apparently secured from its effects, and yet give no evidence of its presence by exciting any perceptible disorder."—We are not perfectly satisfied with this reasoning. The mother and child, though nourished from the same sources, are distinct animals; and though we well know, that persons who have felt the effects of the variolous and some other morbid poisons, will for ever after be found insensible to their effects, yet we have no reason to suppose the effluvia are not absorbed by them, and thrown out again at the common emunctories. But should these effluvia, in the course of circulation, be applied to an animal still susceptible of the disease, there can be no reason, that we know, why that animal should not be affected. In this manner Mr. Hunter conceives a child in utero may be affected by venereal matter received by its mother in actu coitus.

The other case is perhaps more curious, and still more interesting. Whilst small-pox was rife in a village, and two children inoculated in a family, the mother, at that time in the last month of her pregnancy, was vaccinated one day after the inoculation of her children; the vaccination succeeded in the usual form. Five weeks afterwards she was delivered of a female child, which had all the appearance of small-pox eruption in its early stage. This was the 11th of June. On the 19th the infant expired.

There is something peculiarly interesting in this case. Either the effect of small-pox *effluvia* was superseded in the woman by vaccination, as it may be by the common process of small-pox *inoculation*; or the woman received the effluvia in such a state, that it was conveyed to the fœtus at the time when she was rendered unsusceptible of it by the previous vaccination. The latter seems most probable from the date of the different events. Mr. Gervis, who communicated the paper to Dr. Jenner, concludes with the following words.

"In addition to the circumstance of the mother's conveying the variolous infection to her unborn child, without feeling any indisposition from its action on her own constitution, I must remark that there cannot be a stronger proof of the efficacy of vaccine inoculation than this case affords. But, happily, proofs are not wanted, or I could give my testimony to a great extent."

ARTICLE 21.—*Historical Account of Phillip Howorth, a Boy, in whom Signs of Puberty commenced at an early Age.* By ANTHONY WHITE, M. B. Assistant Surgeon to the Westminster Hospital.

This most interesting history, as we remarked of a former article, though highly worthy of record, can furnish no particular practical hints. We therefore announce it, principally, that future inquirers may be directed where to find it.

The child was born at Quebec Mews, Feb. 1806. He appeared large and healthy; his head was covered with hair of a considerable length; the sutures were closed. At seven months he cut the two lower incisors, and in a quick succession twenty teeth appeared, but not in their regular order. At the age of twelve months he showed marks of disease, which by the event proved to be symptomatic of approaching puberty. All the marks of that change were soon apparent, excepting, that only the rudiments of a beard appear, even to the date of the communication; the pubes and scrotum are however covered with black hair. He has already lost one of his upper teeth. We conceive these are only the temporary teeth; but as the author has promised the Society a continued communication of the future progress in his subject, we must wait for that information.

In August 1808, the boy's height was 3 feet 2 inches; his weight 47 pounds; his health perfect;* his habits partaking of the child, from a want probably of the knowledge of any thing beyond the confined circle of his birth; but his temper, though mild, for the most part manly; his appetite voracious; his features particularly expressive; his muscular strength considerable.

On the whole, we think this case resembles most the account contained in one of the early volumes of Dodsley's Annual Register, we believe almost the only one on record, not referred to by the author of the paper.

The volume closes with an account of presents of books received, and the names of the donors. We cannot conclude our remarks without congratulating the public on the accumulation of so many useful facts, heartily wishing the Society may continue equally successful in collecting, and judicious in selecting materials for a future volume.

---

### Edinburgh Journal, No. XIX.

*Medical Report for Nottingham, from March* 1807 *to March* 1808, By James Clarke, M. D. Physician to the General Hospital, and to the Vaccine Institution.

The first case here related is in many respects highly important; we shall therefore transcribe it in the words of the author. The patient was married; had only one child, who was ten years old; found no reason to suspect herself with child; the symptoms were menorrhagia, pain in the abdomen, which was somewhat enlarged; frequent vomiting of food; faintness, and costiveness.

"October 21st. She has been much better, and thought herself nearly well, but was yesterday seized with pain much resembling labour. The surgeon being sent for, arrived after she had, as the attendants said, miscarried. On careful examination, no embryo was discovered;

"* Minime præter eundum est, quod hic puer virilis, manstupratione interdum utitur. Semen tali modo paratum, ipse ego bis vidi; et, me judice, perfectum et bene elaboratum evadit.—This has taken place, the mother informs me, from the completion of the second year."

discovered; but the discharge consisted of two quarts of hydatids, mixed with pulp like albumen, and a little blood; the hydatids were semipellucid vesicles, about the size of hazel-nuts, connected together by small necks, like bunches of grapes. She has this morning passed some more of the same nature, but nothing like an embryo. Two days since she passed, with a gush of blood, a mass about the size of the palm of the hand, which from her description was albumen. She can this morning turn in bed without any pain, and feels very pleasant and light; the abdomen has sunk to its natural size; her gums and mouth are affected by the mercury; for the last two days she has experienced frequent desire to pass urine, of which she has voided more than usual. Belly regular; pulse 96, regular, and moderately full; some thirst. The medicines omitted; an aperient to be taken immediately, a saline mixture in the day, and a gargle to be used for the mouth.

"23d. Feels some pain in the hypogastric region; belly costive; pulse full, and rather hard; slept ill; no appetite; some fever; passes plenty of urine; slight red discharge; tongue clean; breasts much enlarged, painful, and full of milk; they were yesterday drawn by a child.

"Continue the medicines. Repeat the purgative.

"25th. During the operation of the purgatives some more hydatids were passed. Free from pain in the abdomen; some pain in the head; red discharge continues; pulse rather quick and sharp; urine plentiful; appetite improved; less milk in the breasts; has walked out to-day."

The patient gradually recovered. We recollect another instance, not indeed in all, but in many respects similar. A patient in St. Bartholomew's, soon after weaning her child, and the apparent cessation of any secretion of milk, had many symptoms of pregnancy; her breasts swelled and secreted milk. This continued till she was relieved of what is usually termed a false conception; after which the breasts ceased to secrete for a day or two, and then recommenced with the same regularity as if the patient had been delivered of a child.

Sympathy of the breasts with the uterus is so general, that we cannot be surprised at many of the changes we see taking place under different affections of that organ, nor can we be too cautious in our decision on many suspicious cases.

The next case is no otherwise remarkable than as furnishing another instance of the importance of early blood-letting; as is too often the case, the patient and his friends had done all the mischief they could before the faculty were consulted.

On the succeeding case we must make the same remark. On this occasion we cannot help requesting one important piece of information of Dr. Clarke. What is the condition of the inhabitants of Nottingham and its environs? What is the situation of the manufacturers? Are they better fed and lodged than some other manufacturers? As far as we can perceive by his reports, his patients tients are much affected with those diseases which arise from plethora, and we hear comparatively little of scrofula, or that low fever, of which Dr. Wilson speaks, among the manufacturers at Worcester, which never will bear the lancet. Does this arise from better food, better ventilation, or a more northern latitude?

Even a paralytic case, in the old and feeble, seemed more relieved by purgative remedies than by any other means. At last, it probably was, in great measure, superseded by a smart paroxysm of fever attended with diarrhœa. That convulsions in children, especially if attended with costiveness, should yield to purgatives, is what might be expected in most classes of life.

The candour with which Dr. Clarke relates every part of his practice, induces us to offer a suggestion or two on the following case.

" *Chorea.* The symptoms of this disease often shew much variety, but the one about to be detailed presents much novelty.

" Miss N.——, æt. 19. December 1st, 1807. Was yesterday evening suddenly seized with a constant and involuntary motion of the lower jaw, as if in the act of mastication, attended with much oppression of the præcordia, and convulsive respiration, sometimes with a sense of suffocation, which is immediately excited upon any attempt, however slight, to swallow liquids or solids, which, as soon as they touch the fauces, excite a complete and violent paroxysm of this spasmodic affection, but wholly free from pain. Pulse 88, moderately full, and perfectly regular; belly regular; catamenia regular.

" Knows no other cause for this complaint than fatigue, and close confinement to a sedentary occupation. About nine months since, she experienced a slight attack of the same affection, which continued two days, and suddenly left her without any medical assistance; she has always enjoyed good health.

" She was ordered this morning fifty drops of the tincture of opium, for a dose; about an hour afterwards, five grains of calomel, followed by a dose of common purging mixture; and in six hours after that, she took thirty drops more of the tincture of opium, in a cordial draught.

" 2d. Purgative operated freely; slept well last night, and free from spasm. The motion of the jaw is not so violent this morning; but the head, arms, and legs, have been much agitated, as in chorea; has not taken any nourishment without inducing the suffocating sensation; convulsive respiration, or catching of breath, and oppression of the præcordia undiminished; great hunger and thirst, but fears to take any food; pulse 88, regular; feels no pain at any time. During the slight intermissions, she is in good spirits, and apparently free from disease.

" ℞. Assafœtidæ gr. xv. Mist. camphor. ℥x. Spirit. æther. nitros. gutt. 40. Tinct. opii gutt. 30. m. f. Haustus 4ta. quaque hora sumendus.

" ℞. Opii

"℞. Opii pulv. gr. iv. Ol. cinnam. gutt. j. Mucilag. q. s. ut, f. pil. Hac nocte sumenda et cras mane repetenda.

"3d. Took two of the draughts, and the pill at night and this morning; slept well in the night, and has continued all the day strongly under the influence of the opium; has not experienced any spasm during the whole of to-day; jaw free from agitation; no difficulty or fear in taking food; no remains of convulsive respiration. The disease has moved into the arms, which are at times violently convulsed, but quite still during sleep; the convulsion is much increased on waking, particularly if roused by any noise. Pulse natural; free from pain; no stool since yesterday.

"To drink lemon-juice; the opium pill to be repeated at bed-time, and the purging mixture in the morning.

"4th. Involuntary motion continues in the arms and hands; not so constant, but more violent, *and not diminished in bed;* slept well; pulse natural; takes food freely; belly costive. Repeat the purgative.

"℞. Ammoniareti cupri. Opii aa gr. jss. Conserv. q. s. ut, f. pil. bis indie sumenda.

"Repeat the opium pill at bed-time.

"5th. The purgative operated freely; spasms less violent, and chiefly in the arms; sometimes the breathing is affected, but that very slightly; pulse natural; spirits good; urinary secretion has been in every respect natural. Continue the medicines.

"8th. The involuntary motion has been for the last two days entirely confined to the head, very slight, and at times totally suspended, particularly after a strong paroxysm; pulse natural; appetite good; has taken two of the day-pills, by mistake, for one dose; has been sick every night; belly very costive; took some castor oil this morning. Omit the pills.

"℞. Ammoniareti cupri gr. j. Opii gr. jss. Conserv. q. s. ut. f. pil. bis indie sumenda.

"Repeat the opiate at bed-time.

"12th. Catamenia appeared on the 9th, before the usual period; the sickness still continuing, she has omitted the day-pills; has experienced very little involuntary motion, but yesterday evening she had a very severe attack, followed by a restless night, with horrid dreams, and repeated startings; she has at present much involuntary motion of the head, and some affection of the voice; spirits very much depressed, and the mind very anxious; appetite indifferent; catamenia absent; belly costive.

"Has taken of calomel and gamboge, each five grains, this morning. To repeat the opiate pill at bed-time.

"15th. Has continued much better; some pain in the bowels, which are constipated; much sickness after her medicines. The opium has been omitted, and one grain of hyoscyamus substituted for it, but, from the increasing nausea, it also was discontinued.

 "℞. Ex-

"℞. Extracti gentian. Carbon. ferri præcip. Pil. aloes c. myrrha aa ʒj. M. f. pil. 36, quarum sumat iij. bis indie.

"Repeat the purging mixture.

"18th. Has continued nearly free from involuntary motion: general health appears good; countenance healthy; belly regular; sleeps well; appetite moderate; wishes to visit her relatives. To continue the pills, and to take occasionally the purging mixture.

"January 30th, 1808. Has returned two or three days. During her absence, she experienced a return of the spasms: the medical attendant prescribed small doses of opium, and the arsenical solution. She complains of much soreness in the throat and stomach, with griping pains; she has lost flesh, and feels feeble; countenance sickly. This evening, without any known cause, the spasms returned with their former violence, attended with a sense of suffocation; strong involuntary motion of the arms, and some action of the lower jaw, with repeated sighing; pulse very feeble; belly regular; catamenia regular. To take some purging mixture immediately; three grains of opium, with one drop of oil of cinnamon at bed-time.

"8th. The opium has been gradually diminished to one grain, combined with two grains of hyoscyamus; she has taken a febrifuge medicine in the day; the paroxysms of the disease have been severe, but terminating in sleep; although violent, they have been less frequent; and, for the last two days, she has been wholly free from them. She is much debilitated, and complains of weakness in her right leg, which, it has been lately observed, she draws after her when walking. The opium was entirely omitted one night, but from an accidental cause; a paroxysm was induced, which continued violent the greater part of the night, and even strong during sleep; the soreness and griping pain have been removed. Belly, which has been very lax, is now regular; appetite improved; good spirits; she can now write, which she has not accomplished since her first attack.

"To continue the opiate and the febrifuge.

"March 26th. During the period since the last report, she has continued her opium at times; and vegetable tonics, as colombo, gentian, &c. have been substituted for the febrifuge; a blister has been applied to the side, to relieve pain in that part; has for some days had pain in the head; countenance flushed; pulse full; great heat of the forehead.

"Leeches were applied to the temples, and a strict vegetable diet recommended.

"℞. Pil. galb. comp ʒiſs. Pil. aloes, c myrrh. ʒſs. M. f. pil. 24 sumat iij. bis indie.

"November 25th. Since the last report, she has taken the fetid pills occasionally, and used the cold shower-bath twice in the week, during the whole summer; she has repeatedly lost her voice during two or three days. The paroxysms of disease to which she has has been subject, have been purely hysterical, nothing like the former complaint; she has persevered in the vegetable diet: the pains in the head and side were soon removed; her countenance and general appearance is perfectly healthy; she has gained flesh and strength. She has this evening felt nausea; unpleasant taste, with a load at stomach, for which she is to take an emetic.

"30th. A severe paroxysm of fever came on yesterday, without any known cause; belly regular.

"To repeat the febrifuge medicine.

"February, 1809. The febrile affection, mentioned in the last report, subsisted about ten days, since which she has remained in every respect in most perfect health, and has again returned to common diet.

"The motion of the lower jaw was very rapid and violent, not to be restrained by holding the jaw. The convulsive respiration, or catching of the breath, strongly resembled the hydrophobia symptom, as noted in Hill's case, in this Number; the sensation of suffocation was extremely distressing, and in the first two or three days, produced even by the sight of food. During the whole of the paroxysm she never experienced any pain, but always complained of fatigue in the parts affected; the pulse never was affected, either during the paroxysm, or afterwards. The catamenia were, in every respect, natural. Her employment was book-keeping, at which she was sometimes necessarily confined; her temperament was nearly sanguine; her disposition particularly amiable. She experienced great inconvenience in not being able to take food; hunger and thirst became almost insupportable. The slightest sudden movement, noise, or stream of cold air, to which she had the greatest dislike, produced great agitation. When the involuntary motion was confined to the head, it often resembled the agitation of paralytics; the feces were generally nearly natural, perfectly so in respect to colour. The report of the 30th January mentions much constitutional affection; certainly an unexpected change had taken place in a few days, which she attributed to the arsenical solution. In February, the paroxysms terminated in sleep; this, with the other symptoms before noticed, gave some apprehension of a conversion of disease to epilepsy, which was prevented only by the plan adopted. She did not experience merely a loss of voice, but often of the power of speech, and sometimes even of the power of moving the lips, which, though unattended with spasms, were generally consequent on a paroxysm. When this and other hysterical symptoms appeared, the cold-shower-bath was added to the former treatment. The violent paroxysm of fever in November must be considered a salutary action, and may be supposed to have removed the original disease.

"There are many things remarkable in this case, particularly the symptoms so strongly resembling hydrophobia. There was the same abhorrence of food, not from inability to swallow, but from the suffocation such an attempt produced. The effect that a stream of of cold air produced was similar to that in hydrophobia. Sufficient food is now offered for the speculative theorist."

Our speculations on the subject shall be very concise. If this complaint arose from fatigue and close confinement to a sedentary employment; if about nine months before she experienced similar symptoms in a slighter degree, and those symptoms suddenly left her without any medical assistance, we do not perceive, by the account, the necessity of doses, which to us appear very powerful, both in opium, calomel, and gamboge. Should the young lady be again afflicted in a similar manner, might it not be safe to trust a little more to nature, when so little was gained by art.

A case of Epilepsia, in a young woman of about three months standing, was cured under the use of purgative remedies with tonics, and at one time cold bathing.

A case of Diabetes, treated according to Dr. Rollo's plan, without advantage, has been much relieved by Mr. Wat's treatment, but the issue not yet ascertained.

Dyspnœa Aquosa. This is a case of that species of bronchitis, which Dr. Badham has distinguished by the term asthenica.

"Mary Wilford, æt. 56, married, spinner, came under the care of the Reporter, as an out-patient, February 23d, 1808. Complaining of much pain in the head, particularly over the orbits; much cough, which returns by fits, but irregularly attended with dyspnœa; stricture across the chest, without pain; cannot lie down in bed; feet and body swell towards night; fevered; pulse feeble and intermitting; urine scanty; belly costive.

"Has been subject to asthenica."

This we conceive must be a misprint of *asthma* or *asthenia*. If the latter, we wish a word more adapted to common understandings had been used.

We believe no one questions that purgative medicines will promote absorption.

A case of incontinence of urine yielded to tinct. of cantharides, in those moderate doses of twelve drops, to which we were accustomed, but which would appear nothing in the hands of bolder practitioners.

The report closes with a curious case of fractured vertebra.

"A man fell backwards from the threshold of a workshop, about fourteen feet from the ground: he was taken up in a state of insensibility, and carried home. In a little time he was able to point out the seat of injury; but an attentive examination did not enable the surgeon to discover the nature of the accident. He complained of much pain when any pressure was made on the back of the neck; the lower extremities were paralytic; the stools and urine passed involuntarily; and he died on the fourteenth day after the accident.

"Dissection.

"The vertebral column of the neck, dissected from the muscles, was carefully removed from the body. A fracture was immediately discovered, discovered, which took its course through the spinous process, and completely through the body of the fifth vertebra; the lateral portions of the vertebra being sawed away, shewed distinctly the internal view of the fracture. It then appeared evident that, upon the slightest motion, the sharp edges of the fracture must have pressed upon the medulla spinalis. There was no dislocation, nor the slightest displacement of bones, the fracture being perfectly simple, without the smallest spicula of bone. The sheath of the medulla spinalis was laid open the whole extent; at the seat of injury it contained much pus, which had proceeded up to the seat of the fourth vertebra, but there was not the least appearance of injury of the medulla below the seat of fracture. Much coagulated blood was formed under the integuments covering the fifth vertebra. The reporter very much regrets that he had not an opportunity of procuring a more detailed account of the symptoms that ensued from this injury."

Would it not in all such cases be desirable, during life, to attempt cutting down to the vertebræ, in order to ascertain their situation. In this instance the matter was seated within the *theca*, which it might not be safe to cut into; but, perhaps, an early attention to the parts might have prevented the suppuration. We offer this only as a suggestion, well aware of the hazard attending such an operation.

ARTICLE 2.—*Case of Hydrophobia, with an Account of the Appearances on Dissection.* By HENRY OLDKNOW, Surgeon, Nottingham.

IN this case, which adds another to the dreary catalogue, the patient was bitten in the scrotum, and the dog's tooth had penetrated deep into one finger of the left hand. The wound in the scrotum was pared and causticated. That in the finger was only cauterized. Whether amputation of the latter would have secured the patient we cannot say, but when the symptoms occurred, they were attended with pain in the hand only.

Tracheotomy was performed, and though without success, yet it may be useful to record that it was unattended with danger.

On examining the body after death, inflammation appeared on the mucous membrane of the trachæa, extending from the glottis to the subdivisions of the bronchiæ, with considerable thick mucous secretion; considerable erythematous inflammation about the cardiac part of the stomach.

"Another case of the hydrophobia," says our author, "has lately occurred in a village near Nottingham, any particulars of which I am not in possession of, except that the disease occurred about a period of six weeks after the bite, and was extremely rapid in its progress. I mention it because the dog that bit this individual belonged to a man in this town, and was not suspected to be mad until ten days after he had bitten this unfortunate sufferer. He eat and drank heartily, shewed no signs of indisposition, hunted as usual, and occasionally went into a neighbour's house, among children, children, without injuring any one of them; but on the morning of the tenth day, he was observed in the street, snapping at every dog that passed, and was immediately taken in and destroyed.

"This is a very curious and important circumstance, inasmuch as it tends to shew, that canine madness, in its incipient stages, is very difficult of detection; and that a dog, at this period of the disorder, is capable of communicating the infection, which, I believe, is not generally undertsood."

ARTICLE 3.—*Case of Aphonia cured by Purgatives.* By RICHARD JONES, M. D. Surgeon, 2d. Dragoons.

This paper contains a case of Aphonia, and another of H steria, cured by purgatives. It should be added, that one of the patients was habitually costive, and the other had just arrived from a voyage of twenty-eight days, during which he had not had an evacuation by the rectum.

ARTICLE 4.—*Case of Purulent Ophthalmia occurring in an aged Person, with Remarks on the Origin of that Disease.* By WILLIAM SIMMONS, Surgeon, of Manchester.

This paper shews that purulent Ophthalmia may arise without the cause ascribed by Mr. Ware; but we cannot well suppose, that in Mr. Ware's extensive practice he has not often traced it from the cause to which he ascribes it.

ARTICLE 5.—*Discrepance of Opinion in Two of Dr. Wilson's Papers, pointed out.* By JAMES WOODHAM, Esq. London.

This paper being of itself a *review*, it dces not become us to make any further remarks on the subject.

ARTICLE 6.—*Observations on the Treatment of Diabetes.* By ROBERT WATT, Surgeon, Paisley.

To this paper Mr. Watt attaches the following motto. "Does not the cure of some diseases depend upon the same principle as the suspension in cure of gonorrhœa by a fever." JOHN HUNTER.

The paper abounds with good sense. As it is in some measure controversial, we shall not engage in it any further than to show the general drift of Mr. Watts's argument. The cure of diabetes and other chronic diseases by depletion, he seems to impute to that restorative power which the constitution takes up after being much reduced by an acute disease, or from any other cause; and conceives, that to such as dislike the constant repetition of animal food, such a diet may prove as little nutritive as a confinement to vegetable matter.

ARTICLE 7.—*Two Cases of Carditis.* By W. HENCHMAN CROWFOOT, Esq. Member of the Royal College of Surgeons.

These cases are well worth recording, inasmuch as they show how much more frequent inflammation of the heart and its membranes occurs than is by some suspected.

The first was unfortunately not seen by Mr. Crowfoot, till the symptoms symptoms had continued too long to admit of any assistance. It should also be remarked, that the patient had suffered by a similar complaint two or three years before, from the effects of which he had never perfectly recovered.

"On opening the thorax, the pericardium was found *closely adhering* to the *whole* surface of the heart, the apex of which was, by that means, confined down to the diaphragm; a considerable portion of ossific matter was deposited between the two lamina of the pericardium, and every part of the heart afforded marks of most severe inflammation."

Is it not probable, that some of this disorganization may have been the effect of the first inflammation, and have laid the foundation of that subsequent ill health of which the patient complained, previous to her last attack.

The second case is peculiarly interesting, from the connection which has been lately remarked between rheumatism and inflammation of the heart.*

The writer remarks, that all the usual remedies for acute rheumatism were used, except bleeding. "Towards the end of January," says he, "the pain in the side, attended with great distress also at the heart, became the most troublesome symptoms, but seemed to alternate with the extremities." The nature of the disease, however, it is candidly acknowledged, was not suspected till the patient, naturally of a weak constitution, was too much reduced to bear bleeding. We submit it to the author, whether in the advanced stage of a future case, which must otherwise terminate fatally, this *remedium anceps* would not be justifiable.

The great good sense and candour which mark the character of the author in every part of his paper, render any apology on our part as unnecessary, as it would be improper to urge our opinion any further.

ARTICLE 8.—*Account of some Experiments relating to certain Opinions of Bichât, communicated in a Letter from Dr.* PHILLIP WILSON *to Dr.* ANDREW DUNCAN, Jun.

We have often had occasion to admire the ingenuity of some people in making a plain thing obscure, and then developing this obscurity in such a manner as to fancy themselves, and sometimes persuade others, that they have made a discovery. This kind of reasoning very much resembles those ingenious puzzles invented by grown people for the amusement of children.

"A herring and half for three-halfpence, how many will there be for eleven-pence?" Had the question been, A herring for one penny, how many will be for sixpence? The answer would have been too easy to have led the mind into any labyrinth, and both the proposer and solver would have lost their claim to any merit.

Dr. Wil-

* See our account of Mr. Dundas's paper in Medico-Chirurgical Memoirs.

Dr. Wilson is accused of having adopted some of Bichât's opinions, without acknowledging their source. First, let us see what these opinions are, and whether they are worth quarrelling about, before we determine who had the claim to priority.

" The following, says Dr. Wilson, are the opinions which I am accused of borrowing from Bichât.

" 1st. That the brain exercises no direct controul over the motion of the heart, or other muscles of involuntary motion.

" 2d. That the death of the brain occasions that of the heart, by interrupting the breathing, which it does by destroying the powers of the diaphragm, and intercostal muscles.

3d. " That the functions of life may be divided into organic and animal; that is, in more common language, vital and animal; of which the latter are subject to constant intermissions and renewal, their organs requiring intervals of rest; the former incessant, their organs being endowed with an excitability, which is never impaired, except by disease or death.

" 4th. That organic contractility, that is, vital excitability, has no relation to a centre, but exists in every part, independently of its existence in any other, while animal contractility, that is, animal excitability, in every part, depends on the existence of a common centre.

" 5th. That respiration is placed, as it were, between what Bichât calls organic and animal life, that is, between the two sets of functions, the vital and animal; partaking of the former in as far as it influences the state and circulation of the blood in the lungs; and of the latter, in as far as it is performed by muscles wholly of voluntary motion; the intercostal muscles and diaphragm."

" These opinions," say Dr. W. " were published by Bichât, in the year 1799.

" The first, namely, that the brain exercises no direct influence over the motion of the heart, &c. *I* endeavoured to defend, in a paper presented to the Royal Medical Society of Edinburgh, in the year 1791 or 1792, I forget which. The paper is divided into two parts, the first is wholly occupied by arguments in favour of this opinion."

If the paper is a long one, it must have required great ingenuity to find a long string of arguments to prove what every one may see or feel whenever he pleases, and what was thought so simple by one, who left nothing unproved by experiment, which required proof, that he only mentions it in answer to some unfounded opinions which had prevailed concerning the cause of the blood's motion.

" The heart's *motion*," says Mr. Hunter, (Treatise on the Blood, p. 148, " does not arise from an immediate impulse from the brain, as it does in the voluntary muscles."

After this, we shall not trouble our readers with the sagacious experiments Dr. Wilson undertook, to show that such an affection of of the brain as suspended all voluntary motion, produced no effect on the heart. It is a pity he had not seen some *dead drunk* sailor, in whom, though most of the voluntary actions are suspended, yet the heart is little altered in its actions; the rabits might then have been spared.

"The second opinion, namely, that the death of the brain occasions the death of the heart, by interrupting the breathing, which it does by destroying the powers of the diaphragm and intercostal muscles, *I* also stated," says Dr. Wilson, "in the paper presented to the Royal Medical Society, and shall beg leave to copy verbatim what I said of it in a course of lectures which I gave in Edinburgh in 1796. 'Why does the motion of the heart continue in apoplexy, while that of almost all the other muscles of the body is lost? Most cases of apoplexy arise from the application of mechanical or chemical agents to the nervous system. But it would be easy to adduce experiments, capable of proving that mechanical, and, as far as they have been tried, chemical agents, applied to the brain, although they affect violently the muscles of voluntary motion, are incapable of deranging the motions of the heart. But why should the motion of the heart be instantly destroyed in certain cases of apoplexy produced by a more powerful cause than usual? It would not be difficult to show, that the muscles of inspiration are altogether muscles of voluntary motion, and, consequently, that they must cease to act when the powers of the nervous system are destroyed. But when they cease to perform their office, the blood no longer undergoes the usual change in the lungs, which is necessary for the continuance of the heart's motion."

The nearest dependance of the heart, says Mr. Hunter, (Treatise on the Blood, p. 150) is on the lungs; the stoppage of respiration produces a stoppage of circulation, or the heart's motion. And in the preceding page: "We have as regularly the stimulus for respiration; the moment one is finished, an immediate demand taking place; and if prevented, *as this action is under the influence of the will*, the stimulus of want is increased."

Thus, what Dr. Wilson taught in a Course of Lectures, in 1796, we see was published, but not as a discovery, by Mr. Hunter's executor, in 1795; and if Dr. W. had enjoyed the happiness of attending Mr. Hunter's course about twenty years before, he would probably have heard something of the kind as early as that period. We mean something *of the same kind* that Dr. Wilson taught in his lectures, because in them he comes nearer to Mr. Hunter than to Bichât, in his eagerness to claim a priority to whom he falls into an error, which may be pardoned in the French philosopher, but is not so easily excused in a countryman of Mr. Hunter.

Bichât, we see, deals *death* about with as much ease as Buonaparte, and without staying to consider what he is doing. Mr. Hunter, on the contrary, thinks it worth while to tell us what he means by death. Dr. Wilson, in his paper, as far as he quotes from his former writings and lectures, says nothing about *death*, but but speaks only of *motion* or *action*. But after Bichât had so familiarly introduced this gloomy tyrant, the English physician could not be behind hand with him.

"On examining this rabit, (says he, in the paper now before us) 25 minutes after the solution was injected, I found the muscles of voluntary motions flaccid, and the *animal dead*. *The motion*, however, *of the intestines was still strong* in many parts of their track, and continued so for 36 minutes longer, during which I observed it." p. 304.

Now this is one of the most extraordinary phenomena in a *dead animal* we have ever heard of; and the bare mention of it, shows the absolute necessity of determining what we mean when we speak of death.

Let Dr. Wilson beware how he copies *wild theories from a neighbouring nation*. It is impossible to say how far this disorganizing system may extend, if we admit it into physic. In the year 1796, before he was tainted with French principles, we find him reasoning only about the heart's *motion*, but now he tells openly the second opinion, namely, that the *death* of the brain occasions the *death* of the heart, by interrupting the breathing, &c.*

Why did he not refer to what Mr. Hunter had proved by experiment in the passage before quoted? As this work was published a year before Dr. Wilson's lectures, the recollection was probably fresh in his memory at that time, though now obliterated by the flowery language of the French philosopher. If he turns to the passage in Mr. Hunter, he will find that *the stoppage of motion* is by no means synonymous with *death*.

"Thus, says Mr. Hunter, in the passage above cited, "in my experiments on artificial breathing, the heart soon ceased acting, whenever I left off acting with my bellows: and upon renewing my artificial breathing, it, in a very short time, renewed its action."

We shall conclude this remark in the manner we are often obliged to do, by expressing our wish, that whenever philosophers undertake to reason, they would first explain their terms, and then adhere to such explanation. It may be said that every one knows what is meant by death. How then can we say that an animal is dead, when the peristaltic motion continues? By the effect produced on the brain all voluntary motion was suspended, and the muscular rigidity, if universally extended to all the muscles of voluntary motion, must have prevented respiration; but we have no proof that if respiration had been artificially renewed, the action of the heart might not have been recovered.

( To be concluded in our next. )

Report

